# Impact of microRNA Expression in Human Atrial Tissue in Patients with Atrial Fibrillation Undergoing Cardiac Surgery

**DOI:** 10.1371/journal.pone.0073397

**Published:** 2013-09-12

**Authors:** Hiroyuki Nishi, Taichi Sakaguchi, Shigeru Miyagawa, Yasushi Yoshikawa, Satsuki Fukushima, Shunsuke Saito, Takayoshi Ueno, Toru Kuratani, Yoshiki Sawa

**Affiliations:** Department of Cardiovascular Surgery, Osaka University Graduate School of Medicine, Osaka, Japan; Tokai University, Japan

## Abstract

**Background:**

Although microRNA (miRNA) regulates initiation and/or progression of atrial fibrillation (AF) in canine AF models, the underlying mechanism in humans remains unclear. We speculated that certain miRNAs in atrial tissue are related to AF, and evaluated the relationship of miRNA expression in human atrial tissue in cardiac surgery patients.

**Methods:**

Right atrial tissues from 29 patients undergoing cardiovascular surgery were divided into 3 groups [A: chronic AF or unsuccessful maze, n=6; B: successful maze, n=10; C: sinus rhythm (SR) n=13]. miRNA expression was determined using high density microarrays and with Reverse transcriptase-polymerase chain reaction (RT-PCR). Fibrosis was examined using Masson trichrome staining.

**Results:**

miRNA microarray analysis showed elevated miRNA-21, miRNA-23b, miRNA-199b, and miRNA-208b in AF as compared to SR groups. RT-PCR showed elevated miRNA-21 (1.9-fold) and miRNA-208b (4.2-fold) in AF as compared to the SR groups. miRNA-21 expression increased from Group C to A (A: 2.1-fold, B: 1.8-fold, C: 1.0-fold). Fibrosis increased from C to A (A: 43.0±12.9%, B: 21.3±6.1%, C: 11.9±3.1%). Percent fibrosis and miRNA-21 expression were correlated (r=0.508, p<0.05). The plasma levels of miRNA-21 in AF patients was significantly decreased as compared to the healthy volunteers (p<0.05).

**Conclusion:**

The expression of miRNA-21 in human atrial tissue was found to be related to atrial fibrosis and might affect AF occurrence, indicating its usefulness as a biomarker for cardiac surgery management.

## Introduction

Atrial fibrillation (AF), the most common type of arrhythmia, affects 5% of the population older than 65 years old and 7.1% of those older than 85 years old [1]. Despite several basic and clinical studies, the precise underlying mechanism of onset and persistence of AF have not been completely elucidated, because various factors, such as inflammation, atrial fibrosis, oxidative stress injury, systemic inflammatory states, autonomic imbalances, and neurohormonal activation, are considered to be related its occurrence [2,3]. There are several potential structural changes in atrial tissue that affect the occurrence of AF, including dilatation and fibrosis [4]. microRNAs (miRNAs) comprise a broad class of small non-coding RNAs composed of 21-25 ribonucleotides, which control the expression of complementary target messenger RNAs [5-8]. Recently, researchers have begun to investigate the roles of these small non-protein-coding RNAs in the cardiovascular system and currently available results show the potential of miRNAs as a novel mechanism for AF [6,9,10]. However, published studies that focused on miRNAs and AF are sparse, and most investigated using various animal models. As a result, scant information regarding the relationship between miRNA expression in human atrial tissue and occurrence of AF is available.

In cardiovascular surgery, we use AF as a treatment target when attempting restore sinus rhythm with a maze procedure, which, initially described by Cox and colleagues [11,12] has been proven to be safe and effective for surgical treatment of AF. However, its success rate remains 70-90% and more appropriate biomarkers to predict the success rate of a maze procedure are needed. We speculated that certain miRNAs in atrial tissue may be related to AF occurrence, which would indicate their use as biomarkers for cardiac surgery management. We evaluated the relationships of miRNA expressions in human atrial tissue with AF after cardiac surgery and normal sinus rhythm at the time of cardiac surgery.

## Methods

The protocols of this study were approved by the ethics committees of Osaka University Hospital and the patients provided written informed consent before enrollment. This investigation complied with the principles that govern the use of human tissues outlined in the Declaration of Helsinki.

### Patient selection

From December 2010, Right atrial tissues from 29 patients undergoing open heart surgery were obtained. Of those, 4 patients with chronic AF who did not undergo a maze procedure due to various reasons and 12 patients with persistent AF who underwent maze procedure were classified as the preoperative AF group (n=16). Among patients in the preoperative AF group, those who had AF after the maze procedure (unsuccessful maze procedure) and chronic AF without undergoing a maze procedure was classified as Group A (n=6), while those who had a successful maze operation and postoperative SR were classified as Group B (n=10). Thirteen patients with preoperative SR who underwent isolated aortic valve replacement or on-pump beating coronary artery bypass grafting were included in the SR group (Group C). The postoperative status of heart rhythm in patients who underwent a Maze procedure was investigated at discharge and each outpatient follow-up examination. The present results are based on the status at the final examination 6 months after surgery. The baseline characteristics of the patients are listed in Table 1.

**Table 1:** Demographic profile of the subjects.

	Preoperative AF group (n= 16)		SR group (Group C) (n=13)
	Group A (n=6)	Group B (n=10)	
Age (years)	70.0±5.2 (64-75)	72.5±5.0 (63-78)	63.0±10.1 (43-73)
Gender (male/female)	4/2	6/4	9/4
Disease	AS+TR: 2AS+MR+TR:1AS+MR+TR:1 MS+TR:2	AR+MR: 3 MR+TR:3AS+MR+TR:2AR+MS+TR: 1MR:1	AS:4 AR:3 ASR:1 MR:1 CAD:4
Operative procedures	AVR+TAP:2MVR+TAP:2AVR+MAP+TAP:2	AVR+MAP:2 Bentall+MAP:1AVR+MAP+TAP:2AVR+MVR+TAP:1MVP +TAP:3MAP:1	AVR:8 MVP:1 CABG:4
Maze procedure	Yes:1 No:4	Yes 8	No:13
LAD (mm)	54.8±7.6	54.0±7.7	44.0±9.2
LVEF (%)	69.5±7.5	52.7±15.3	62.0±8.6

AS: aortic stenosis, TR: tricuspid regurgitation, AR, aortic regurgitation MR, mitral regurgitation, MS: mitral stenosis, CAD, coronary artery disease, AVR: aortic valve replacement, TAP: tricuspid annuloplasty, MAP: mitral annuloplasty, MVR: mitral vlave replacement, Bentall: Bentall procedure, MVP: mitral valve repair, CABG: coronary artery bypass grafting, LAD: left atrial dimension,LVEF: left ventricular ejection fraction

### Surgical management

At our institution, open heart surgery is generally performed via a median sternotomy with standard techniques for moderate hypothermic cardiopulmonary bypass. Myocardial protection is provided with intermittent antegrade and retrograde cold blood cardioplegia. Postoperatively, atrial and ventricular pacing are done as required for bradycardia. Following surgery, patients are admitted to the intensive care unit and then transferred to a monitored intermediate care unit. While each patient is hospitalized, an alarm-triggered telemetry system is used to continuously monitor arrhythmia and follow-up examinations are continued for the entire hospital stay.

A modified Cox IV procedure was applied for all of the present patients using a bipolar radiofrequency system (Atricure Inc, Westchester, OH) and a cryoablation device (Frigitronics, Cooper Surgical, Shelton, CT) as previously described [11].

### Preparation of human atrial tissue and blood samples

Tissue samples were obtained from the right atrium of the 16 patients with preoperative AF during right atriotomy procedures. These right atrial tissues were considered to be surgical waste. For patients in the SR group, tissue samples were extracted from the venous cannulation site in the right atrium (free wall near atrial appendage).

After placing a purse string suture, we excised the right atrial tissue from each patient, which was also considered to be surgical waste. The samples were divided into 2 specimens, with 1 used for miRNA analysis and the other for histological analysis. The right atrial specimens used for miRNA investigation were submerged in RNA later^TM^ (QIAGEN, Hilden, Germany) and stored at 4°C to stabilize the RNA until miRNA array analysis. Other specimens were preserved in 10% formalin for histological examinations.

Blood samples for miRNA detection were collected from the patients in the operation room. Four healthy volunteers without any evidence of CAD or inflammatory disorders served as control group. Total RNA was enriched from all EDTA-plasma samples using the mirVana^TM^ PARIS RNA isolation kit (Ambion, Austin, TX), according to the manufacturer’s instructions. RNA concentration and purity were evaluated by a spectrophotometer. Aliquots of RNA were stored at -80°C until use.

### RNA isolation and miRNA arrays

The atrial tissue specimens were homogenized using a Tissue Lyser II homogenizer (QIAGEN, Hilden, Germany). Total RNA was extracted from the stored human right atrial homogenized tissue specimens using an mirVana^TM^ miRNA isolation kit (Ambion, Austin, TX), according to the manufacturer’s instructions. RNA concentration and purity were evaluated using a spectrophotometer. Aliquots of RNA were stored at -80°C until use.

Total RNA was reverse-transcribed using Megaplex Primer Pools (Human Pools A v2.1 and B v2.0), and miRNA expression was screened using a TaqMan Human MicroRNA Array A (ABI, Foster City, Calif), according to the manufacturer’s instructions. We used TaqMan Array Human MicroRNA A card (Rot number 4398965, http://www.appliedbiosystems.jp/website/larger?sid=125776 The authors agree to share our data upon request.) QPCR was carried out with an Applied Biosystems 7900HT thermocycler using the manufacturer’s recommended protocol. Detailed analysis of the results was performed using Real-Time Statminer software (Integromics).

### Reverse transcriptase-polymerase chain reaction analysis

Total RNA isolated from the stored specimens was reverse transcribed with QuantiTect Reverse Transcriptase (Qiagen, Hilden, Germany). Reverse transcriptase-polymerase chain reaction (RT-PCR) analysis was performed using a 7500 Fast Real-Time PCR System (Applied Biosystems, Foster City, Calif). Quantitative real-time RT-PCR of miRNAs was performed using TaqMan MicroRNA assays (Applied Biosystems, Foster City, California), according to the manufacturer’s instructions, with a 7500 real-time RT-PCR system (Applied Biosystems, Foster City). Expression levels of the small nuclear RNA, miRNA-16, were used as the normalization control. The threshold cycle (Ct) was defined as the fractional cycle number at which fluorescence exceeded the given threshold. Relative quantifications were calculated with the comparative Ct method (2^-ΔΔCt^).

### Histological analysis

Following fixation in 4% paraformaldehyde in phosphate buffered saline, the tissues were dehydrated in graded alcohol and embedded in paraffin. Deparaffinized sections were stained with hematoxylin and eosin (H&E), while Masson Trichrome staining was also performed. Each slide was examined by light microscopy (Keyence Corp., Osaka, Japan) and microscopic images were obtained, then transferred to a personal computer. Assessment of atrial fibrosis was performed with Masson Trichrome staining at ×100 magnification. The percentage of total fibrotic area, as determined by Masson’s trichrome staining, was calculated by image analysis of the sections using a planimetry method with Windows MetaMorph software (Universal Imaging Corporation, Downingtown, PA). The fibrosis area appeared as yellow following Masson Trichrome staining. The extent of fibrosis in each field was defined as the percent area of fibrosis. Each sample was divided into 10 fields and the percent fibrosis value for each was added to obtain the total area of fibrosis area per sample. The percent fibrosis area was calculated using the average of the 10 fields.

### Statistics

Data were analyzed using the Statview 5.0 software program (SAS Institute Inc., Cary, NC). Results are expressed as the mean ± standard deviation. A Mann-Whitney U-test was used for comparison of continuous variables and Fisher’s exact test for comparison of frequencies between 2 groups. Correlations between variables were tested with Spearman’s rank correlation analysis. A p value less than 0.05 was used to select variables to enter in the multivariate model. Statistical significance was also defined by a p value of 0.05 or less. An miRNA expression heat map was constructed by unsupervised hierarchical clustering of miRNAs.

## Results

### Surgical outcomes and patient characteristics

In 12 patients who underwent a maze procedure, 10 (83%) achieved postoperative SR conversion. There were no significant differences between successful and unsuccessful maze procedure cases in regard to age (72.5 years vs. 74.0 years), left ventricular ejection fraction (53% vs. 75%), prevalence of the valvular disease, and left atrium dimension (54 mm vs. 57 mm).

### Microarray analysis of miRNA expression

**Figure 1:**
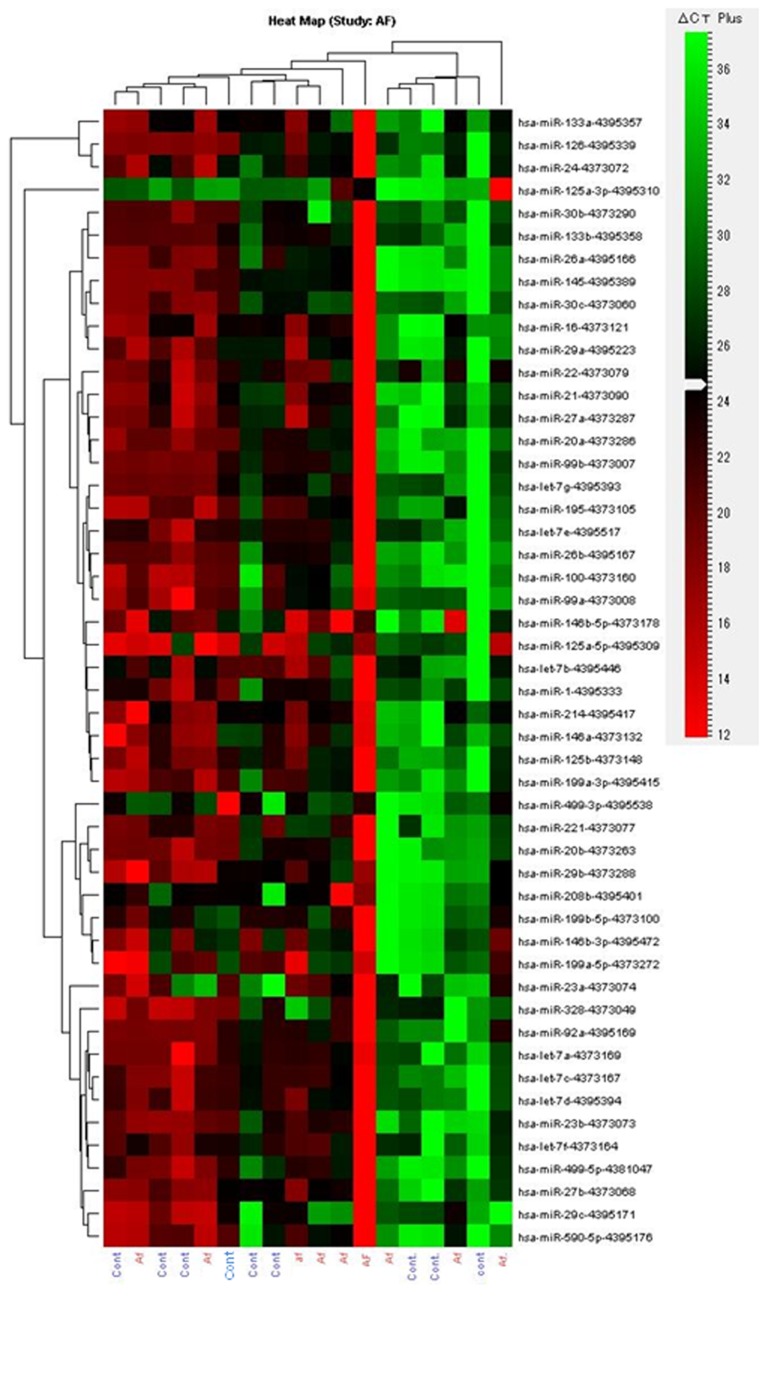
Heat maps of the Microarray analysis of miRNA. The miRNA microarray cluster of atrial fibrillation patients and sinus rhythm patients clustered into 2 distinct sets according to their diagnosis based on signal strength. Sample names are indicated at the bottom and each miRNA is indicated on the right side.

The results showed that 98 miRNAs were expressed differently in the preoperative AF group as compared with the SR group (http://dx.doi.org/10.5061/dryad.8p1t6), of which 94 were upregulated and 4 downregulated in the preoperative AF group (Table S1). Figure 1 presents a heat map of expressions of the top 20 most abundantly expressed miRNAs in the heart specimens and 9 miRNAs reported to be related to occurrence of AF in previous studies. Of these, miRNA-21 (3.3-fold), miRNA-23b (4.7-fold), miRNA-199b (3.3-fold), and miRNA-208b (3.1-fold) were highly elevated preoperatively in the AF group as compared to the SR group.

### RT-PCR analysis of miRNA expression

**Figure 2:**
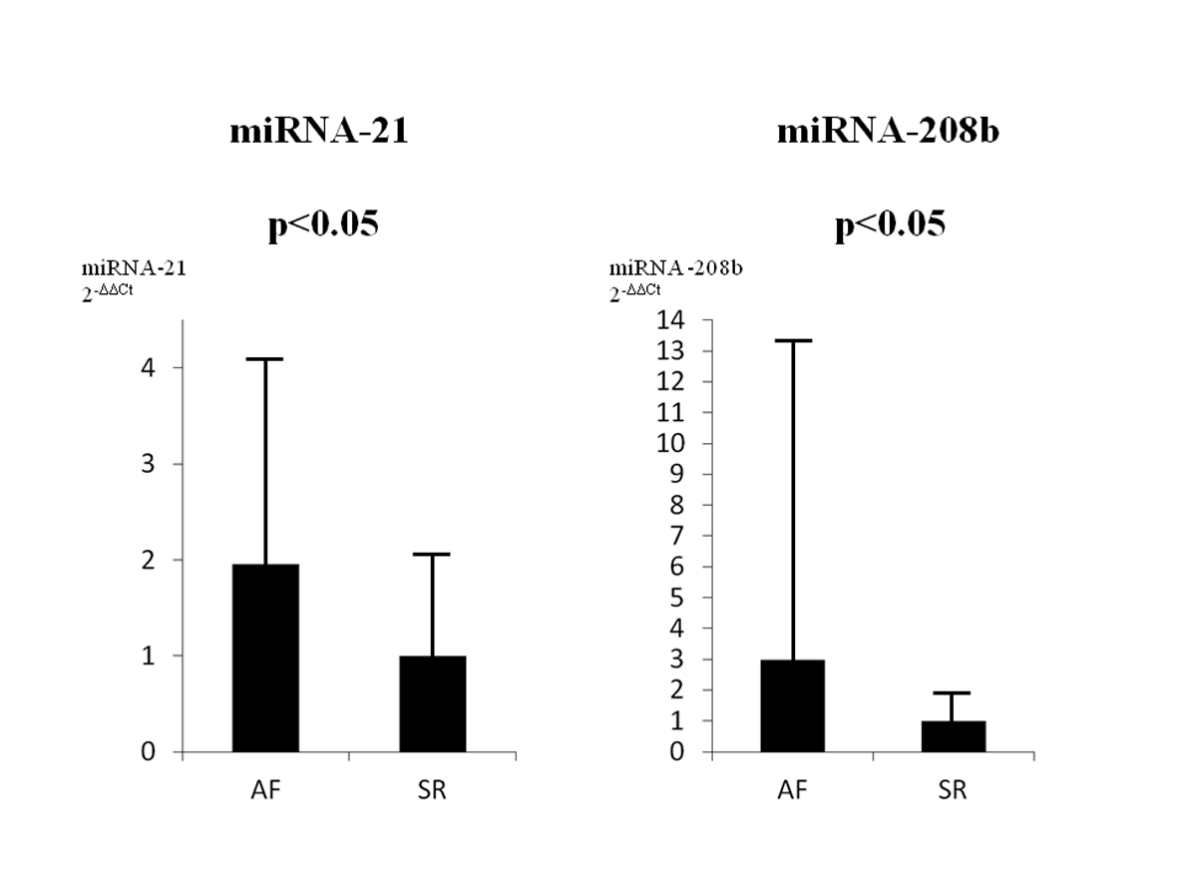
Reverse transcriptase-polymerase chain reaction analysis showed high expressions of miRNA-21 (1.9 fold) and 208b (4.2 fold) in the atrial fibrillation (AF) group as compared to the sinus rhythm (SR) group.

Independent RNA samples that were prepared in parallel with the samples for the microarray were used for quantitative real-time RT-PCR analysis. miRNAs previously described to be related to the heart or AF (miRNA-1, 21, 22, 23, 24, 26, 29,30, 125, 133, 146, 195, 199, 208, 214, 221, 328, 499, 590, and let 7f) were investigated in all of the specimens (Table S2). There was no significant difference for miRNA-23b and miRNA-199b, while quantitative real-time RT-PCR analysis showed high expressions of miRNA-21 (1.9-fold) and miRNA-208b (3.0-fold) in the preoperative AF group as compared to the SR group (Figure 2).

### miRNA expression and surgical outcome

**Figure 3:**
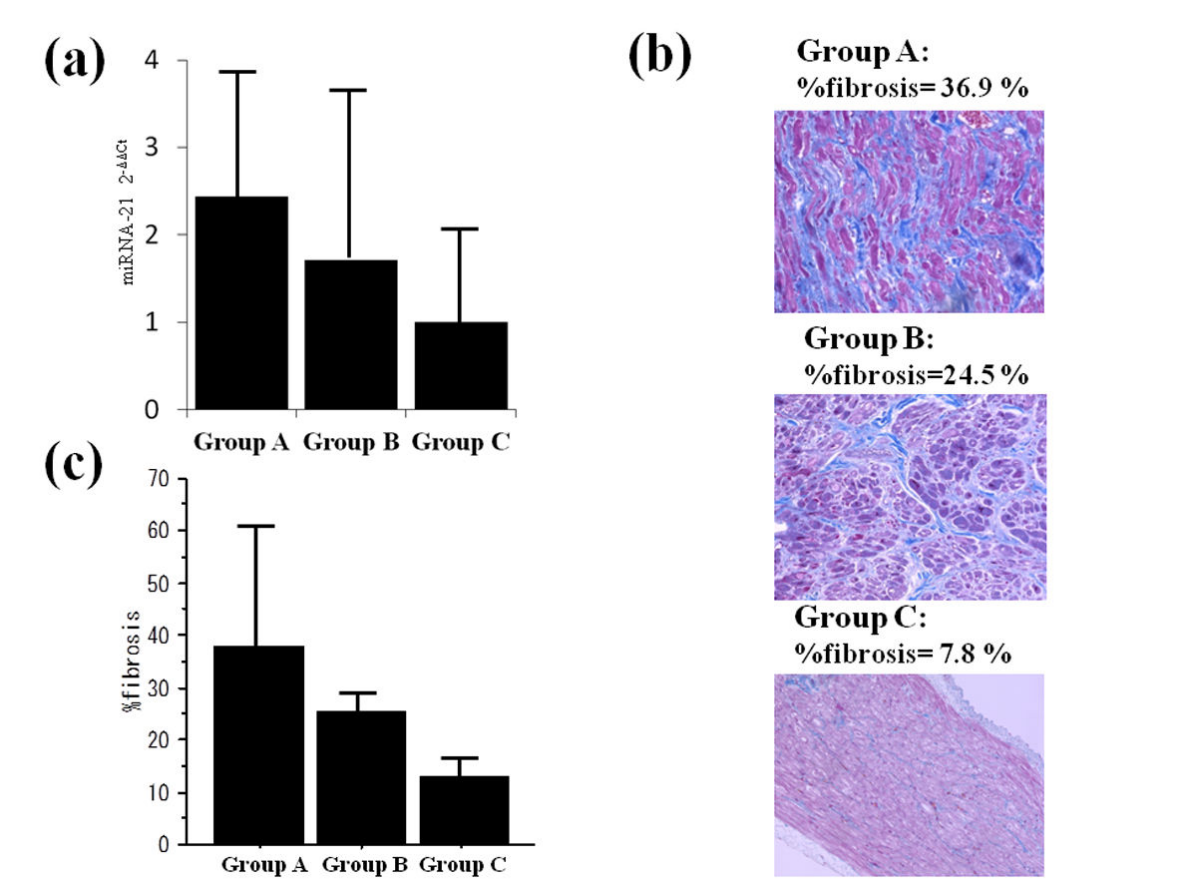
In altered expression profiles of microRNAs in each group (A,B) relative to group C, miRNA-21expression was increased from Group C to A (a). % fibrosis of representative cases in each group (**b**). % fibrosis and surgical outcome: Fibrosis was increased from group C to A (A: 43.0±12.9%, B: 21.3±6.1%, C: 11.9±3.1%). (**c)**.

To analyze the relationship between miRNA expression and surgical outcomes, we compared miRNA expressions, with the SR group patients that did not develop postoperative AF used as a control group. In altered expression profiles of miRNAs in each group (A, B) relative to group C, miRNA 21 expression showed the greatest increase in Group A, followed in order by Groups B and C (A: 2.4-fold, B:1.7-fold, C: 1.0-fold; Figure 3a).

### Fibrosis and surgical outcomes

The mean fibrosis area in patients in the preoperative AF group was 29.6±14.1%, which was significantly larger than that in those in the SR group (11.9±3.1%, p<0.05, example: Figure 3b). Overall, percent fibrosis was greatest in Group A, followed in order by Groups B, and C (A: 43.0±12.9%, B: 21.3±6.1%, C: 11.9±3.1%, Figure 3c). There were significant differences between patients with and without a successful maze procedure (p<0.05).

### Relationship between miRNA expression and fibrosis

**Figure 4:**
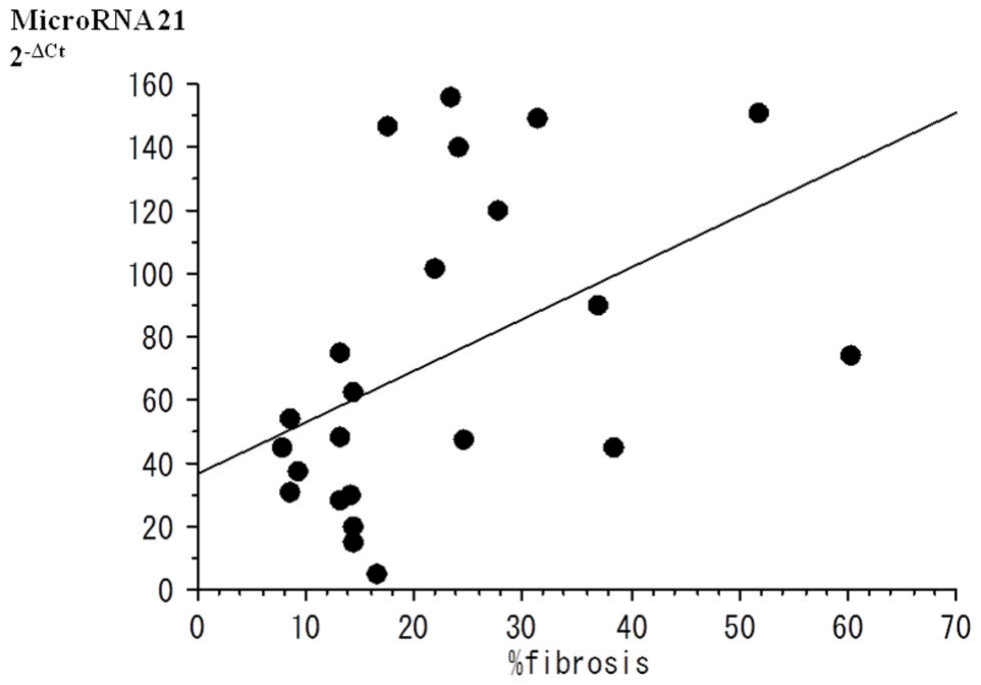
There was a correlation between % fibrosis and miRNA-21expression (r=0.462, p<0.05).

Several expressions of miRNAs were compared to percent fibrosis. Although no significant relationship of miRNA-208b expression with percent fibrosis was found, there was a correlation between percent fibrosis and miRNA-21 expression (r=0.462, p<0.05; Figure 4).

### miRNA-21 expression in blood samples (Figure 5)

The plasma levels of miRNA-21 in patients with AF was significantly decreased as compared to the healthy volunteers (p<0.05). There was an inverse relationship between the level of miRNA-21 expression in atrial tissue and plasma level.

**Figure 5:**
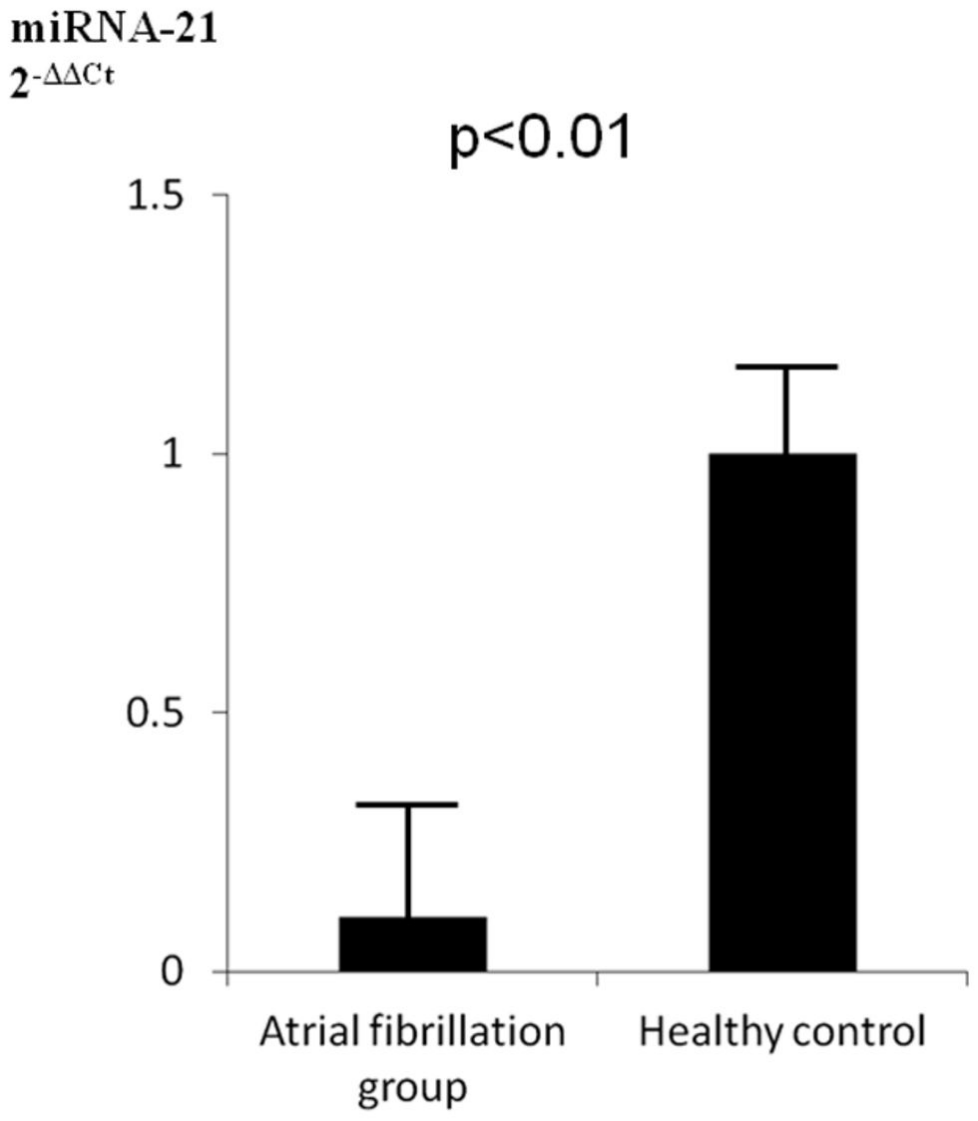
Reverse transcriptase-polymerase chain reaction analysis of the blood samples showed low expressions of miRNA-21 in the atrial fibrillation group as compared to the healthy volunteer group.

## Discussion

Our findings revealed miRNA expression signatures in right atrial tissues of patients with AF and SR. There were 4 differently expressed miRNAs among cardiac- and AF-related miRNAs in the preoperative AF patients, as compared with the SR patients. Furthermore, we compared the expressions of miRNAs shown to be upregulated in RT-PCR analysis with maze procedure outcome. Interestingly, miRNA-21 expression was highest in patients with chronic AF or an unsuccessful maze procedure, and gradually decreased in order from those with a successful maze procedure to SR patients. Furthermore, a positive relationship between the extent of fibrosis and expression level of miRNA-21 in atrial tissue was detected. This study is one of the initial reports of the potential association of miRNA-21 in human atrial tissue, as well as the relationship of miRNA-21 expression with atrial fibrosis and cardiac surgery outcome [13,14]. Although there is a growing body of evidence indicating that a specially altered pattern of miRNA expression is associated with AF, the relationship between miRNAs and AF is still unknown, and scant information is available regarding their expressions in human tissues [5,9,10,15-20]. With this in mind, the present results provide information regarding the potential association of miRNAs in patients with AF.

Recently published studies focusing on miRNAs and AF have demonstrated the potential of miRNAs in a novel mechanism for AF [5,9,10]. One report revealed that miRNA-26 is significantly down-regulated in AF, which results in an increase of *Ik1* density to repress *KCNJ2* [5]. A very recent study demonstrated repression of IcaL by miRNA-328 as an independent mechanism for AF in dog models as well as human atrial samples from AF patients with rheumatic heart disease [9]. In the present study, we did not detect significant differences between AF and SR patients regarding the expressions of miRNA-26, and miRNA-328. This contradictory finding may reflect the different settings of our study. MiRNA-26, and 328 are reported to contribute to AF via various ion channels, all of which have been detected in AF animal models. This setting is a relatively acute stage and may be different from a clinical setting, such as in the present cases. Thus, patients with chronic AF may no longer have those changes via ion channels.

MiRNAs are also considered to be related with regulation of fibrosis and apoptosis of cardiomyocytes [16-18]. One study revealed that down-regulation of miRNA-133 promotes AF through a mechanism favoring atrial structural remodeling [16]. They concluded that the pro-fibrotic response to nicotine in the canine atrium is critically dependent upon down-regulation of the anti-fibrotic miRNA-133 and miRNA-590. In addition to these miRNAs, several others have also been reported to regulate fibrogenesis in cardiac tissues. For example, miRNA-208 and 21 have been characterized as pro-fibrotic, and implicated in cardiac hypertrophy/heart failure and myocardial infarction, respectively [17,18].

The present findings revealed that miRNA-21 and miRNA-208 are highly expressed in patients with AF, though there were no significant differences between AF and SR patients regarding miRNA-133 and miRNA-590 expressions. These results imply that pro-fibrotic factors are more important than inhibition of anti-fibrotic factors in clinical settings similar to our study. MiRNA-21was reported to regulate the ERK-MAP kinase signaling pathway in cardiac fibroblasts [18]. Increased miRNA-21 promotes fibroblast survival and growth factor secretion, and also controls the extent of interstitial fibrosis, while such an increase may also reflect the number of fibroblasts. On the other hand, Patrick et al. reported that miRNA-21was not required for cardiac fibrosis in response to acute or chronic injury in mice [19]. However, their suggested mechanism was based on ventricle tissue. From the results of our study showing that miRNA-21expression level was positively correlated with the amount of fibrosis in the right atrium, fibroblast activation or the number of fibroblasts may reflect the presence of fibrosis in human atrial tissue, which is correlated with surgical outcome.

Atrial fibrosis is a common feature of clinical AF and several studies have noted AF in various clinical settings [20-23]. Increased collagen deposition has been documented in patients with AF as compared with SR control subjects [20], while another report noted that the extent of atrial fibrosis by magnetic resonance imaging predicts AF recurrence after AF ablation. In the field of cardiac surgery, Nakai et al. reported that among patients undergoing coronary artery bypass grafting, those with postoperative AF tended to have an increased amount of atrial fibrosis [22]. Another study revealed that left atrial histological features such as fibrosis were more extensive in those with an unsuccessful maze procedure as compared to those with a successful maze procedure [23]. Consistent with these previous studies, the present results showed a relationship between the extent of atrial fibrosis and maze procedure success.

Our findings suggest a new therapeutic entry point for AF and may assist establishment of an miRNA therapeutic intervention strategy in a setting of cardiovascular disease. If prediction of the extent of atrial fibrosis could be made based on the plasma level of miRNA-21, that would provide useful information for deciding treatment for AF, such as whether a maze procedure should be performed or the timing of initiation of medical therapy, as well as others. With this in mind, we checked the plasma levels of miRNA-21 and compared them to the levels in healthy volunteers. That level in patients with AF was significantly decreased as compared to the healthy volunteers. In addition, there was an inverse relationship between the level of expression in atrial tissue and plasma level. This finding was different from a previous study, which found no significant differences between the plasma level of miRNA-21 and AF occurrence [24]. We consider that this discrepancy was due to the different patient backgrounds, as our patients were candidates for surgery, thus the severity of AF may have differed from that of the subjects in the other study. Additional investigation is needed to clarify this issue. Nevertheless, even though it is important to consider various factors, our finding may also contribute to a future clinical application.

### Limitations

It should be noted that even though our study identified miRNA-21 and miRNA-208b as possible important factors in patients with AF in cardiac surgical settings, it does not exclude other mechanisms previously described. In addition, we used maze procedure outcome as a marker for degree of AF, as we considered that patients who had undergone a successful maze procedure had less advanced AF. The main purpose of the present study was not to clarify risk factors of a maze procedure.

Furthermore, the number of subjects in each subgroup was small, thus it is possible important and causative variables were not accounted for in the microarray analyses. In addition, several potential limitations exist in this study, including the small number of patients, and further studies with more patients are necessary to confirm our results. Second, the present study was performed with native human tissues. We did not perform experiments to modulate miRNA levels, thus the findings presented here are indirect and the exact pathological role remains unknown. Additional investigation is needed to clarify the exact mechanism, except for atrial fibrosis. The main purpose of the present study was to determine the relationship between miRNA and AF, thus the findings presented are important in this field of research.

Third, we examined only right atrial free wall tissue and did not assess other parts of the atria, including the left atrium. However, a biopsy of the left atrium is invasive and the procedure itself may result in atrial irritation. We consider that the present evaluation was acceptable due to a previous study that demonstrated histological changes including fibrosis occurring to the same extent in both atria [25]. Finally, the expressions of miRNAs in the atria of our patients would be more apparent if we could use normal human tissues as a control. However, it is impossible to obtain normal human atrial tissues. Additional studies are needed to clarify this issue.

## Conclusions

We found that miRNA-21 expression in human atrial tissue is potentially associated with atrial fibrosis and might affect AF occurrence, which would indicate its potential usefulness as a biomarker for cardiac surgery management. The amount of fibrosis might be also related to the occurrence of AF in cardiac surgery, while a positive relationship between the amount of fibrosis and expression level of miRNA-21 was found. This miRNA expression revealed in human atrial tissue in this study may highlight potential targets for future clinical applications.

## Supporting Information

Table S1 **Microarray expression of microRNAs in patients with atrial fibrillation (AF) compared to those with sinus rhythm (SR): (Fold differences > 2.0).**
(DOC)

Table S2 **RT-PCR analysis of microRNAs in patients with atrial fibrillation compared to those with sinus rhythm.**
(DOC)
