# Supplementary material for: Impact of microRNA Expression in Human Atrial Tissue in Patients with Atrial Fibrillation Undergoing Cardiac Surgery
Source: PLoS One. 2013 Sep 12;8(9):e73397. doi: 10.1371/journal.pone.0073397 (PMC3772020; doi:10.1371/journal.pone.0073397)
Supplement: Table S1 — Microarray expression of microRNAs in patients with atrial fibrillation (AF) compared to those with sinus rhythm (SR): (Fold differences > 2.0). (DOC) [file pone.0073397.s001.doc]

**Table S1**

**Microarray expression of micro RNAs in patients with atrial fibrillation (AF) compared to those with sinus rhythm (SR): (Fold differences > 2.0)**

| micro RNA | AF (2^-ΔCt^ ) | SR (2^-ΔCt^) | Fold differences | Regulation |
| --- | --- | --- | --- | --- |
| hsa-let-7a | 8.5E-05 | 3.82019E-05 | 2.22614 | up |
| hsa-let-7d | 9.11E-05 | 3.90749E-05 | 2.33153 | up |
| hsa-let-7f | 6.1E-05 | 1.30074E-05 | 4.6918 | up |
| hsa-miR-101 | 1.25E-05 | 2.63334E-06 | 4.73911 | up |
| hsa-miR-103 | 9.55E-05 | 4.24407E-05 | 2.2496 | up |
| hsa-miR-106b | 6.4E-05 | 3.0077E-05 | 2.12636 | up |
| hsa-miR-125b | 0.001618 | 0.000741298 | 2.18276 | up |
| hsa-miR-127-3p | 1.51E-05 | 6.86468E-06 | 2.19926 | up |
| hsa-miR-129-3p | 1.32E-06 | 2.99428E-07 | 4.39409 | up |
| hsa-miR-130a | 4.24E-05 | 1.60834E-05 | 2.63758 | up |
| hsa-miR-130b | 1.19E-05 | 4.04684E-06 | 2.93879 | up |
| hsa-miR-134 | 4.51E-05 | 1.50926E-05 | 2.98799 | up |
| hsa-miR-140-5p | 0.000582 | 0.000262548 | 2.21809 | up |
| hsa-miR-142-3p | 6.79E-05 | 2.0927E-05 | 3.24457 | up |
| hsa-miR-146b | 0.001545 | 0.00057552 | 2.68398 | up |
| hsa-miR-148b | 9.5E-06 | 2.21329E-06 | 4.29056 | up |
| hsa-miR-152 | 0.000271 | 0.00012075 | 2.24549 | up |
| hsa-miR-15a | 9.29E-06 | 3.42056E-06 | 2.71511 | up |
| hsa-miR-15b | 6.71E-05 | 3.22112E-05 | 2.0833 | up |
| hsa-miR-181a | 5.99E-05 | 2.71031E-05 | 2.20926 | up |
| hsa-miR-181c | 2.91E-06 | 1.4289E-06 | 2.03483 | up |
| hsa-miR-184 | 1.52E-05 | 2.0447E-06 | 7.44106 | up |
| hsa-miR-185 | 2.85E-05 | 9.78842E-06 | 2.91207 | up |
| hsa-miR-187 | 0.000303 | 0.000130192 | 2.32532 | up |
| hsa-miR-190 | 2.11E-06 | 7.7956E-07 | 2.70846 | up |
| hsa-miR-193a-3p | 1.13E-05 | 5.28849E-06 | 2.14212 | up |
| hsa-miR-196b | 1.33E-06 | 5.51905E-07 | 2.40514 | up |
| hsa-miR-199a-5p | 1.04E-06 | 5.02441E-07 | 2.07963 | up |
| hsa-miR-199b-5p | 1.99E-06 | 6.01206E-07 | 3.30387 | up |
| hsa-miR-203 | 3.59E-05 | 1.63657E-05 | 2.19513 | up |
| hsa-miR-208b | 2.83E-06 | 9.22367E-07 | 3.06516 | up |
| hsa-miR-20b | 1.28E-05 | 6.32372E-06 | 2.02395 | up |
| hsa-miR-210 | 0.000404 | 0.000199897 | 2.0188 | up |
| hsa-miR-21 | 0.000304 | 9.24439E-05 | 3.28726 | up |
| hsa-miR-215 | 4.13E-07 | 1.93571E-07 | 2.13292 | up |
| hsa-miR-216a | 7E-07 | 1.93571E-07 | 3.61508 | up |
| hsa-miR-216b | 5.22E-07 | 1.93571E-07 | 2.6961 | up |
| hsa-miR-217 | 6.24E-07 | 1.93571E-07 | 3.22367 | up |
| hsa-miR-22 | 0.000562 | 3.55045E-05 | 15.827 | up |
| hsa-miR-23b | 4.49E-05 | 9.51289E-06 | 4.71675 | up |
| hsa-miR-24 | 0.081998 | 0.036298847 | 2.25898 | up |
| hsa-miR-27a | 0.000461 | 0.000158006 | 2.91772 | up |
| hsa-miR-27b | 0.000143 | 5.03103E-05 | 2.84899 | up |
| hsa-miR-28-5p | 8.64E-05 | 4.27704E-05 | 2.02092 | up |
| hsa-miR-320 | 0.003464 | 0.00162002 | 2.13835 | up |
| hsa-miR-32 | 2.36E-06 | 9.17733E-07 | 2.5704 | up |
| hsa-miR-324-5p | 9.5E-06 | 3.46358E-06 | 2.74187 | up |
| hsa-miR-330-3p | 1.25E-05 | 2.86584E-06 | 4.36126 | up |
| hsa-miR-337-5p | 1.12E-06 | 5.53162E-07 | 2.01606 | up |
| hsa-miR-34a | 3.56E-05 | 1.77735E-05 | 2.00152 | up |
| hsa-miR-361-5p | 6.31E-06 | 1.85524E-06 | 3.39887 | up |
| hsa-miR-362-5p | 1.79E-05 | 7.45746E-06 | 2.4051 | up |
| hsa-miR-371-3p | 5.84E-07 | 2.74638E-07 | 2.12654 | up |
| hsa-miR-372 | 2.54E-05 | 8.9245E-06 | 2.84375 | up |
| hsa-miR-423-5p | 3.15E-06 | 7.76045E-07 | 4.05786 | up |
| hsa-miR-424 | 8.31E-07 | 3.26186E-07 | 2.54667 | up |
| hsa-miR-431 | 4.33E-07 | 1.93571E-07 | 2.23528 | up |
| hsa-miR-449a | 1.17E-06 | 3.57893E-07 | 3.26072 | up |
| hsa-miR-450a | 3.92E-07 | 1.93571E-07 | 2.02357 | up |
| hsa-miR-455-5p | 1.13E-05 | 3.4078E-06 | 3.31739 | up |
| hsa-miR-487a | 6.6E-07 | 1.93571E-07 | 3.41069 | up |
| hsa-miR-487b | 5.97E-06 | 2.2231E-06 | 2.68746 | up |
| hsa-miR-494 | 1.18E-05 | 5.74658E-06 | 2.05416 | up |
| hsa-miR-495 | 5.02E-06 | 2.43975E-06 | 2.0588 | up |
| hsa-miR-499-5p | 4.6E-05 | 2.1667E-05 | 2.12178 | up |
| hsa-miR-500 | 3.74E-06 | 1.70838E-06 | 2.18813 | up |
| hsa-miR-504 | 5.22E-07 | 1.93571E-07 | 2.69648 | up |
| hsa-miR-505 | 5.21E-07 | 1.93571E-07 | 2.68907 | up |
| hsa-miR-508-3p | 5.54E-07 | 1.93571E-07 | 2.86457 | up |
| hsa-miR-509-5p | 1.11E-06 | 4.21924E-07 | 2.63606 | up |
| hsa-miR-511 | 3E-06 | 1.4523E-06 | 2.06724 | up |
| hsa-miR-517a | 2.12E-06 | 8.46418E-07 | 2.49908 | up |
| hsa-miR-517c | 1.98E-06 | 8.46913E-07 | 2.33335 | up |
| hsa-miR-518b | 1.05E-06 | 5.07386E-07 | 2.07314 | up |
| hsa-miR-518f | 6.19E-07 | 1.93571E-07 | 3.19897 | up |
| hsa-miR-520e | 4.37E-07 | 1.93571E-07 | 2.25824 | up |
| hsa-miR-522 | 6.58E-07 | 1.93571E-07 | 3.39841 | up |
| hsa-miR-539 | 9.61E-06 | 3.78686E-06 | 2.53664 | up |
| hsa-miR-542-5p | 4.42E-06 | 1.31558E-06 | 3.35633 | up |
| hsa-miR-545 | 2.02E-06 | 7.24273E-07 | 2.78226 | up |
| hsa-miR-548d-5p | 3.91E-07 | 1.93571E-07 | 2.01811 | up |
| hsa-miR-579 | 5.55E-07 | 2.52516E-07 | 2.19618 | up |
| hsa-miR-597 | 5.49E-07 | 1.93571E-07 | 2.83766 | up |
| hsa-miR-618 | 1.4E-06 | 5.08062E-07 | 2.76457 | up |
| hsa-miR-652 | 3.35E-05 | 9.58978E-06 | 3.49744 | up |
| hsa-miR-660 | 0.000108 | 4.60845E-05 | 2.34063 | up |
| hsa-miR-671-3p | 1.88E-06 | 7.15089E-07 | 2.62373 | up |
| hsa-miR-758 | 5.54E-07 | 2.74459E-07 | 2.01732 | up |
| hsa-miR-874 | 1.8E-05 | 6.34433E-06 | 2.83218 | up |
| hsa-miR-886-5p | 7.65E-05 | 3.55035E-05 | 2.15507 | up |
| hsa-miR-887 | 5.96E-06 | 2.42878E-06 | 2.45197 | up |
| hsa-miR-888 | 4.13E-07 | 1.93571E-07 | 2.13397 | up |
| hsa-miR-93 | 8.63E-05 | 3.92775E-05 | 2.19753 | up |
| hsa-miR-95 | 4.76E-05 | 2.29435E-05 | 2.07389 | up |
|  |  |  |  |  |
| hsa-miR-429 | 3.09E-07 | 8.56336E-07 | 0.3606 | down |
| hsa-miR-31 | 1.15E-05 | 2.7409E-05 | 0.41852 | down |
| hsa-miR-200b | 9.19E-07 | 2.01924E-06 | 0.45533 | down |
| hsa-miR-885-5p | 1.11E-06 | 2.43649E-06 | 0.45658 | down |

AF: atrial fibrillation, SR: Sinus rhythm
