# Supplementary material for: Impact of microRNA Expression in Human Atrial Tissue in Patients with Atrial Fibrillation Undergoing Cardiac Surgery
Source: PLoS One. 2013 Sep 12;8(9):e73397. doi: 10.1371/journal.pone.0073397 (PMC3772020; doi:10.1371/journal.pone.0073397)
Supplement: Table S2 — RT-PCR analysis of microRNAs in patients with atrial fibrillation compared to those with sinus rhythm. (DOC) [file pone.0073397.s002.doc]

**Table S2**

**RT-PCR analysis** of micro RNAs in patients with atrial fibrillation compared to those with sinus rhythm

| micro RNA | AF (2^-ΔCt^ ) | SR (2^-ΔCt^) | p-value |
| --- | --- | --- | --- |
| miR-1 | 226.608 | 224.088 | ns |
| miR-21 | 108.134 | 48.7252 | p<0.05 |
| miR-22 | 10.98 | 8.52907 | ns |
| miR-23b | 48.0737 | 44.1427 | ns |
| miR-24 | 421.829 | 241.039 | ns |
| miR-26 | 161.133 | 152.562 | ns |
| miR-29b | 25.0177 | 17.2626 | ns |
| miR-125b | 174.597 | 170.414 | ns |
| miR-133b | 186.205 | 166.546 | ns |
| miR-146a | 5.5495 | 3.12683 | ns |
| miR-195 | 37.8679 | 29.7181 | ns |
| miR-199b | 3.13191 | 1.64334 | ns |
| miR-208b | 2.43659 | 0.54818 | p<0.05 |
| miR-214 | 38.7145 | 29.349 | ns |
| miR-221 | 3.31306 | 1.46431 | ns |
| miR-328 | 40.5209 | 25.6069 | ns |
| miR-590 | 2.62814 | 1.76533 | ns |
| let-7a | 11.3657 | 8.96169 | ns |
